# Water and Blood Repellent Flexible Tubes

**DOI:** 10.1038/s41598-017-16369-3

**Published:** 2017-11-22

**Authors:** Sasha Hoshian, Esko Kankuri, Robin H. A. Ras, Sami Franssila, Ville Jokinen

**Affiliations:** 1 Department of Chemistry and Materials Science Aalto University School of Chemical Engineering, Espoo, Finland; 2 Brigham and Women’s Hospital, Harvard Medical School, Cambridge, MA 02139 USA; 3 Faculty of Medicine, Department of Pharmacology University of Helsinki, Helsinki, Finland; 4 Department of Applied Physics Aalto University School of Science, Espoo, Finland

## Abstract

A top-down scalable method to produce flexible water and blood repellent tubes is introduced. The method is based on replication of overhanging nanostructures from an aluminum tube template to polydimethylsiloxane (PDMS) via atomic layer deposition (ALD) assisted sacrificial etching. The nanostructured PDMS/titania tubes are superhydrophobic with water contact angles 163 ± 1° (advancing) and 157 ± 1° (receding) without any further coating. Droplets are able to slide through a 4 mm (inner diameter) tube with low sliding angles of less than 10° for a 35 µL droplet. The superhydrophobic tube shows up to 5,000 times increase in acceleration of a sliding droplet compared to a control tube depending on the inclination angle. Compared to a free falling droplet, the superhydrophobic tube reduced the acceleration by only 38.55%, as compared to a 99.99% reduction for a control tube. The superhydrophobic tubes are blood repellent. Blood droplets (35 µL) roll through the tubes at 15° sliding angles without leaving a bloodstain. The tube surface is resistant to adhesion of activated platelets unlike planar control titania and smooth PDMS surfaces.

## Introduction

Superhydrophobicity means a very high water contact angle (>150°), low contact angle hysteresis (<10°) and a low sliding angle of a water droplet on a surface^1^. Superhydrophobic surfaces dramatically reduce the friction and energy consumption via flow drag reduction^2–4^. Many reports are of flexible water and blood in superhydrophobic surfaces both in turbulent and laminar flow^5–11^. Surface friction has a large contribution to total resistance in the turbulent state, e.g.; 50% of conventional airplanes and ships, 70% of submarines and 100% of long-distant pipe transportation^12^. Water droplet motion can be controlled using superhydrophobic planar^13–17^ and non-planar^18–21^ surfaces. Wider applicability of superhydrophobic tubes also demands durability. A thick porous layer can produce a thick plastron at the solid-liquid interface which is a key for durable superhydrophobic surfaces immersed in water^1,22,23^. A lot of research has been done on durability of superhydrophobic surfaces^24–30^ but problems such as cost, yield, flexibility and robustness in use remain unsolved. Another problem is that superhydrophobic surfaces mostly repel water and other liquids such as blood remain a challenge. Superhydrophobic surfaces have recently been indicated as potentially anti-thrombic surfaces that not only resist adhesion of blood droplets but also resist the adhesion of platelets^31–35^. Anti-thrombic surfaces and tubes based on superhydrophobicity could offer a way toward progress in the long-standing problem of thrombogenicity of blood contacting medical devices^36–38^.

Superhydrophobic tubes reported so far have been produced either by hydrophobic coating of a conventional tube^11,39^ or by rolling of a superhydrophobic planar surface^6,40,41^. The coating approach has many challenges; 1: The coating procedure inside a tube can be challenging to optimize for high quality coatings, 2: A coating applied inside a tube is mostly not monolithic so its durability is limited, 3: The coatings are mostly fluorine-based with a possibility of contamination of the liquid and other environmental downsides. The biggest disadvantage of the rolling method is the sealing seam which is a defect that can potentially act as a pinning site.

Here we present monolithic and flexible superhydrophobic tubes that repel both water and blood. The fabrication method is based on atomic layer deposition (ALD)-assisted replication molding from nanostructured aluminum tube templates. Template choice allows both inner and outer diameters to be tuned. The nanostructured polydimethylsiloxane (PDMS)/titania tube is water and blood repellent and it displays anti-adhesive properties toward platelets.

## Results and Discussion

### Fabrication of superhydrophobic tubes

**Figure 1:**
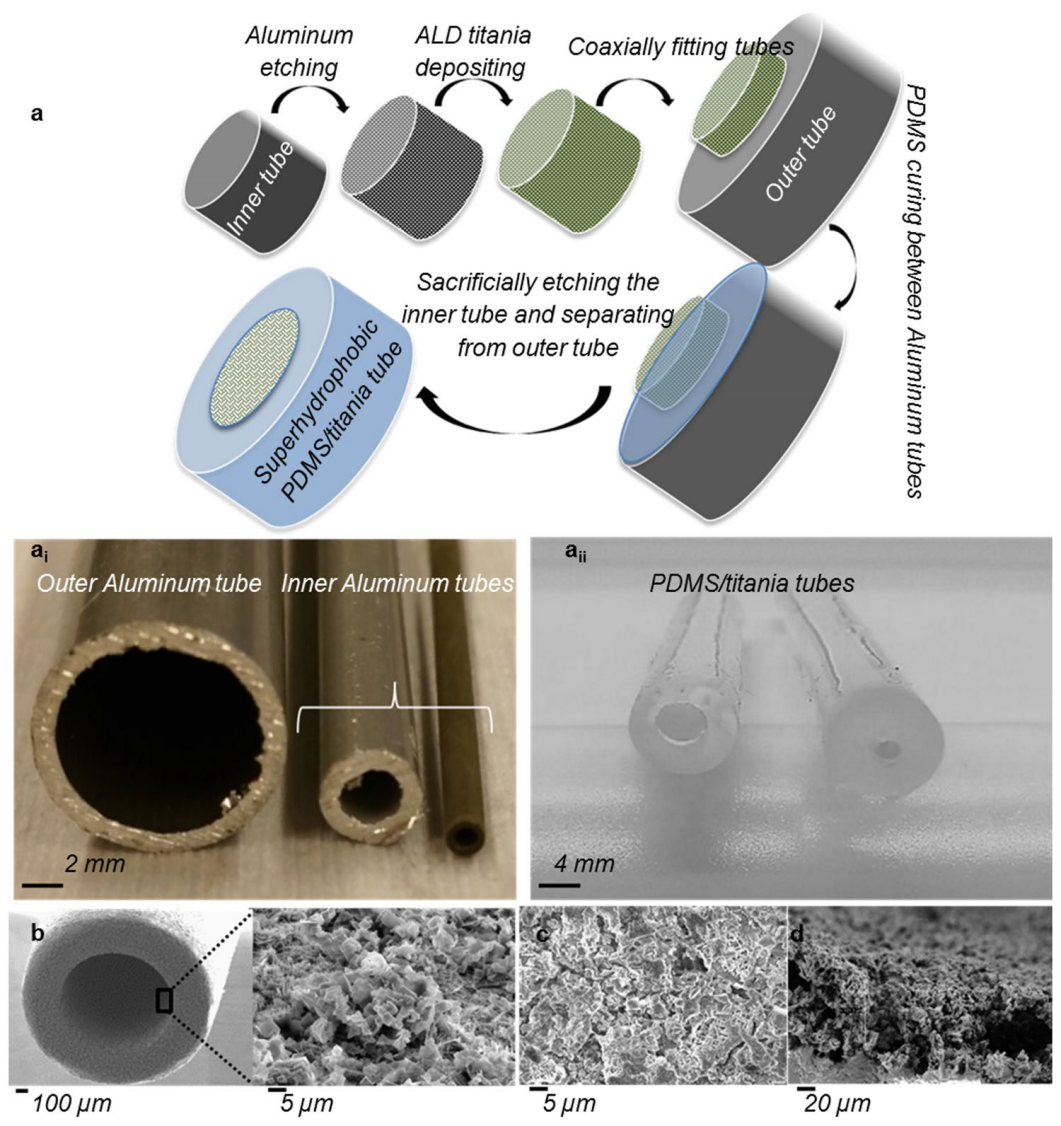
Fabrication process of flexible water and blood repellent tubes. (**a**) Schematic of the fabrication process with corresponding images of templates (a_i_) and replica (a_ii_) tubes. PDMS is cured between two coaxially aligned tubes. Outside of the inner tube was nanostructured by HCl etching and the structures were covered with 20 nm of titania film using ALD. Sacrificial etching of the template was used to release the PDMS/titania tube. SEM micrographs of, (**b**) Template aluminum tubes with scale bar of 100 µm and higher magnification inside the tube with scale bar of 5 µm, (**c**) Finished superhydrophobic PDMS/titania replicas with scale bar of 5 µm, (**d**) Side view of the same sample with scale bar of 20 µm.

The fabrication process of the tubes is illustrated in Figure 1a and it is based on a modified version of our recently reported method for fabricating durable self-healing superhydrophobic hybrid (elastomer/metal-oxide) materials as planar surfaces^42^. In this work, aluminum tubes with 2 mm or 4 mm outer diameter and 10 cm length were chemically etched in 1 M HCl solution for 30 min to get a thick (tens of microns) nanostructured re-entrant porous surface layer (Figure 1b). A 20 nm thick titania film was deposited on the nanostructures using ALD. This tube was then placed coaxially inside another aluminum tube, and the space between the tubes was filled with PDMS. The outer surface of the etched tube thus acted as the template for the inner surface of the polymer replica, shown in Figure 1c,d. Details of fabrication process are given in the *Methods* section. For comparison a control PDMS/titania tube was fabricated using the same method but without structuring the aluminum, resulting in a titania coated PDMS tube with smooth walls. The X-ray photo-electron spectroscopy (XPS) data confirms the existence of titania on PDMS after replication (Supplementary Information and Supplementary Figure S1a).

Both the inner and outer surfaces of the PDMS tubes can be made superhydrophobic independently. The inner surface of the replica tube copies the structure of the outer surface of the inner template tube, while the outer surface of the replica tube copies the structure of the inner surface of the outer template tube. In this work we focused on a tube that is superhydrophobic from the inside. Figure 1b–d shows top and side-view SEM micrograph of the replicated PDMS/titania sample. The side-view SEM confirms the successful replication of 100 µm thickness of hierarchical structures (*l*).

### Water and blood repellency

**Figure 2:**
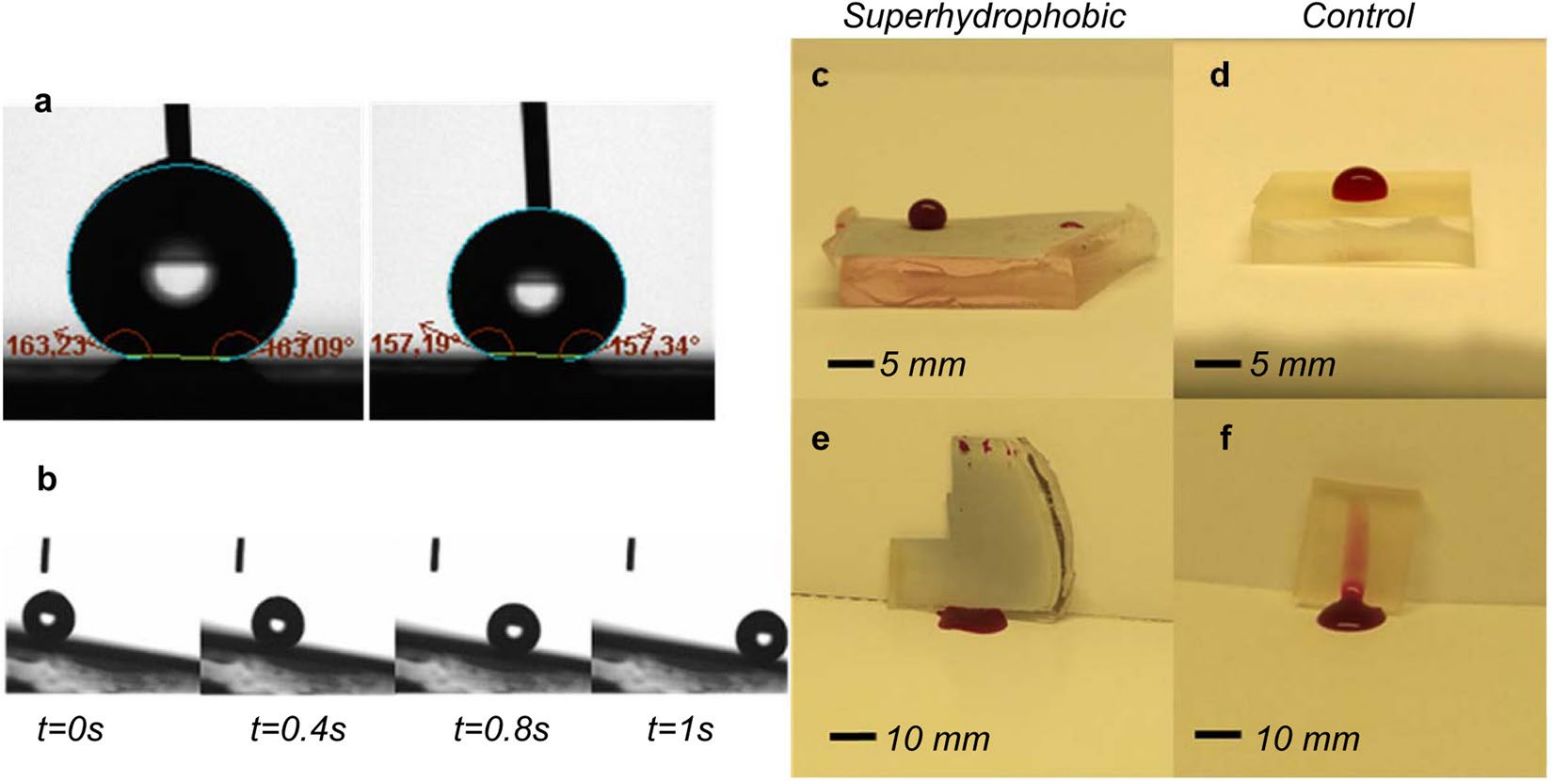
Characterization of water and blood repellency. (**a**) Advancing and receding water contact angle on planar superhydrophobic PDMS/titania sample, (**b**) Snapshots of a 2 µL water droplet rolling off from the surface tilted at 7°. Images of a 35 µL human blood droplet on the, (**c**) superhydrophobic sample, (**d**) on the smooth PDMS/titania control sample, (**e**) Blood droplet sliding off the superhydrophobic PDMS/titania sample without a trace showing the surface is blood repellent, (**f**) Blood droplet sliding off a smooth PDMS/titania control surface leaving a clear trace.

Water contact angles (WCA) of the PDMS/titania samples were measured by the sessile droplet method on planar surfaces fabricated from aluminum plates with exactly the same process as the tubes. Goniometry is not possible for the inner walls of the tubes, but since the same process was used to fabricate the planar references, we assume that the tube walls have the same characteristics. The advancing contact angle for nanostructured PDMS/titania was 163 ± 1° and the receding contact angle was 157 ± 1° (Figure 2a). The sliding angle for 2 µL droplet was less than 10° (Figure 2b). For the smooth PDMS/titania (control) surface the advancing contact angle was 110 ± 1° and the receding contact angle was 90 ± 2°. It is worth mentioning that the nanostructured PDMS surface that was replicated from aluminum template without using ALD titania was not superhydrophobic^42^. The advancing contact angle on nanostructured PDMS surface was 136 ± 1° and the receding contact angle was 100 ± 2°. The SEM images confirm the obvious difference between replicated nanostructures with and without using ALD titania (Supplementary Information and Supplementary Figure S1b,c).

The superhydrophobic PDMS/titania substrates showed also high contact angles with low adhesion to human blood. The static contact angle of a 35 µL blood droplet on the nanostructured PDMS/titania surface was 161 ± 3° (Figure 2c) as compared to 90 ± 2° on the control surface (Figure 2d). Figure 2e,f show a 35 µL blood droplet sliding on both nanostructured PDMS/titania (Figure 2e) and smooth PDMS/titania surface (Figure 2f) tilted to 70°. It is clear from the images that there is no visible trace of blood on the superhydrophobic surface while a large blood trail is left behind on the control surface. The nanostructuring process thus imparts significant blood repellency on the surface and enables blood transportation without macroscopic losses.

### Sliding Droplets inside Tubes

**Figure 3:**
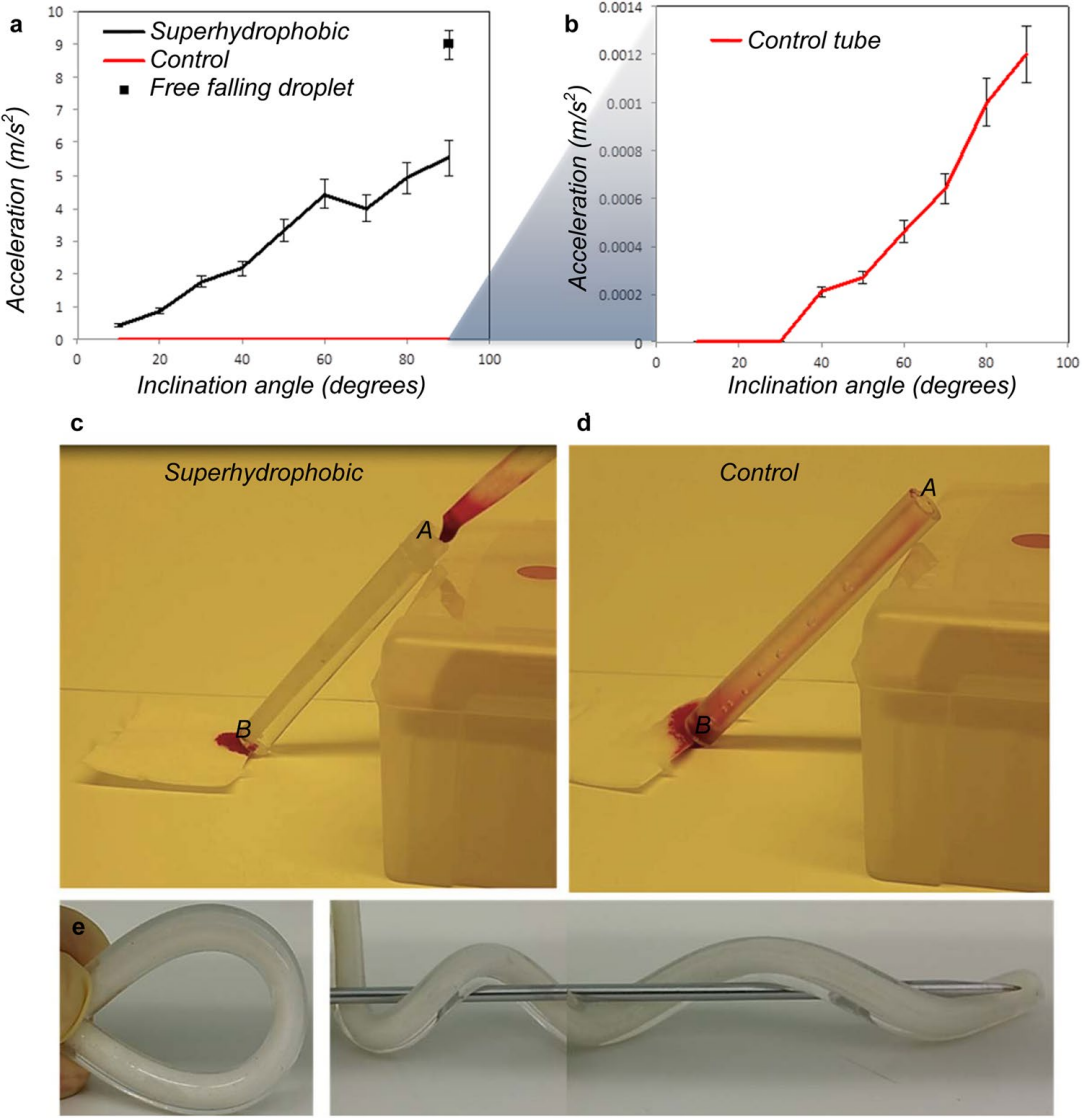
Droplets in flexible superhydrophobic and control tubes. (**a**) Acceleration of a 35 µL sliding water droplet inside superhydrophobic PDMS/titania and (**b**) inside smooth PDMS/titania control tubes with 4 mm inner diameter (the error bars represent s.d. of three measurements), (**c,d**) Images for comparison the transfer of 200 µL human blood in inclination angle Φ = 45° from point A to B in (**c**) superhydrophobic PDMS/titania tube without any visual trace of blood inside the tube and (**d**) smooth PDMS/titania control tube with a clear trace of the blood. (**e**) Flexibility demonstration of the tubes showing they were not broken after several bending and twisting.

The drag reduction of water and blood droplets passing through the tubes was tested by studying the minimum angle that was required for droplet movement and by recording the droplet acceleration as a function of tilting angle (Supplementary Information and Supplementary Figure S2). For these experiments, we utilized a tube with a 4 mm inner diameter. The droplet size was chosen to be 35 µL, which is larger than a sphere with 4 mm diameter, which ensures that the droplet is in contact with the tube from all sides. Figure 3a,b show the acceleration of water droplets in both superhydrophobic tube and in the control tube as a function of the inclination angle (Φ), which was varied from 10° to 90° in 10° increments. The minimum inclination angle for the droplet to slide in the superhydrophobic tube was 10° while for the smooth PDMS/titania control tube it was 40°. For the superhydrophobic tube the accelerations ranged from 0.4–5.5 m/s^2^ as a function of the inclination angle from 10° to 90°, while it ranged from 0–0.001 m/s^2^ for the control tube. The acceleration of a sliding droplet in a 90° inclined (vertical) superhydrophobic tube was 5.5 m/s^2^, which was 5,000 times faster compared with the acceleration of 0,001 m/s^2^ in the control tube. Compared to a free falling droplet in air, the superhydrophobic PDMS/titania tube causes only 39% reduction in the acceleration while the control tube causes 99.99% reduction. The acceleration of a free falling droplet in the same set-up was measured to be 9 m/s^2^, which is close to gravitational acceleration. Video S1 shows the tilted tube measurements.

The acceleration of sliding droplet in an inclined tube depends on the water contact angle and the hysteresis inside the tube. Equation 1 shows the relation between the drag force and contact angle hysteresis in a tube with radius r while γ is the surface tension of the sliding liquid.

1 $${F}_{hysteresis}=2\pi {\rm{r}}\,\gamma ({\cos {\rm{\theta }}}_{{\rm{adv}}}-{\cos {\rm{\theta }}}_{{\rm{rec}}})$$

Calculation from equation 1 indicates one order of magnitude reduction of drag force in superhydrophobic tube compare to the control tube

$$(\frac{{{F}}_{{hysteresis}{(}{control}{)}}}{{{F}}_{{hysteresis}{(}{superhydrophobic}{)}}}=\frac{-0,3094\,{mN}}{-0,0323\,{mN}}\approx 9.55).$$

The total force affecting the droplet is *F*_*gravity*_ + *F*_*hysteresis*_ + *F*_*shear*_. The threshold between a rolling and a pinned droplet can be calculated from the condition *F*_*gravity*_ + *F*_*hysteresis*_ = 0, as the shear component approaches 0 for a barely moving droplet. For the superhydrophobic tube, the gravity force *mg sin(*Φ) equals the hysteresis force when the inclination angle is 5.41° and for the control tube the corresponding threshold inclination angle is 64.32°. These values agree reasonably well with the experiments (Figure 3a,b). In the experiments the droplets in the superhydrophobic tube roll with 10° inclination while droplets in the control tube roll already at inclination angle of 30°, albeit very slowly. The discrepancy in the control tube results could be due to slight differences in the actual contact angles inside the tube compared to the planar surfaces of the same material that were used for the contact angle measurements.

The superhydrophobic tube also reduces the shear forces and viscous drag of liquid flow. We measured the effect of the shear force reduction by measuring the hydraulic resistance (of continuous flow) compared to the control tube (Supplementary Information and Supplementary Figure S3). At low Reynolds numbers, up to 68% and 38% reductions in hydraulic resistance were achieved with superhydrophobic tubes of 2 mm and 4 mm diameters, respectively, compared to a non-superhydrophobic control tube.

The superhydrophobic tube is anti-adhesive toward blood as well as water. After transferring 200 µL of human blood in tubes (10 cm long and 4 mm diameter), no visible trace of blood contamination was detected on the superhydrophobic tube (Figure 3c and Video S2) while in the control tube the blood contamination was fully visible with naked eyes (Figure 3d and Video S2).

The flexibility of the tube is shown in Figure 3e. Bending and releasing of tubes for 50 times was used to test the durability of the superhydrophobic inner surfaces. After this treatment, the average acceleration for a 35 µL droplet in inclined angles (10° to 90°) remained unchanged.

**Figure 4:**
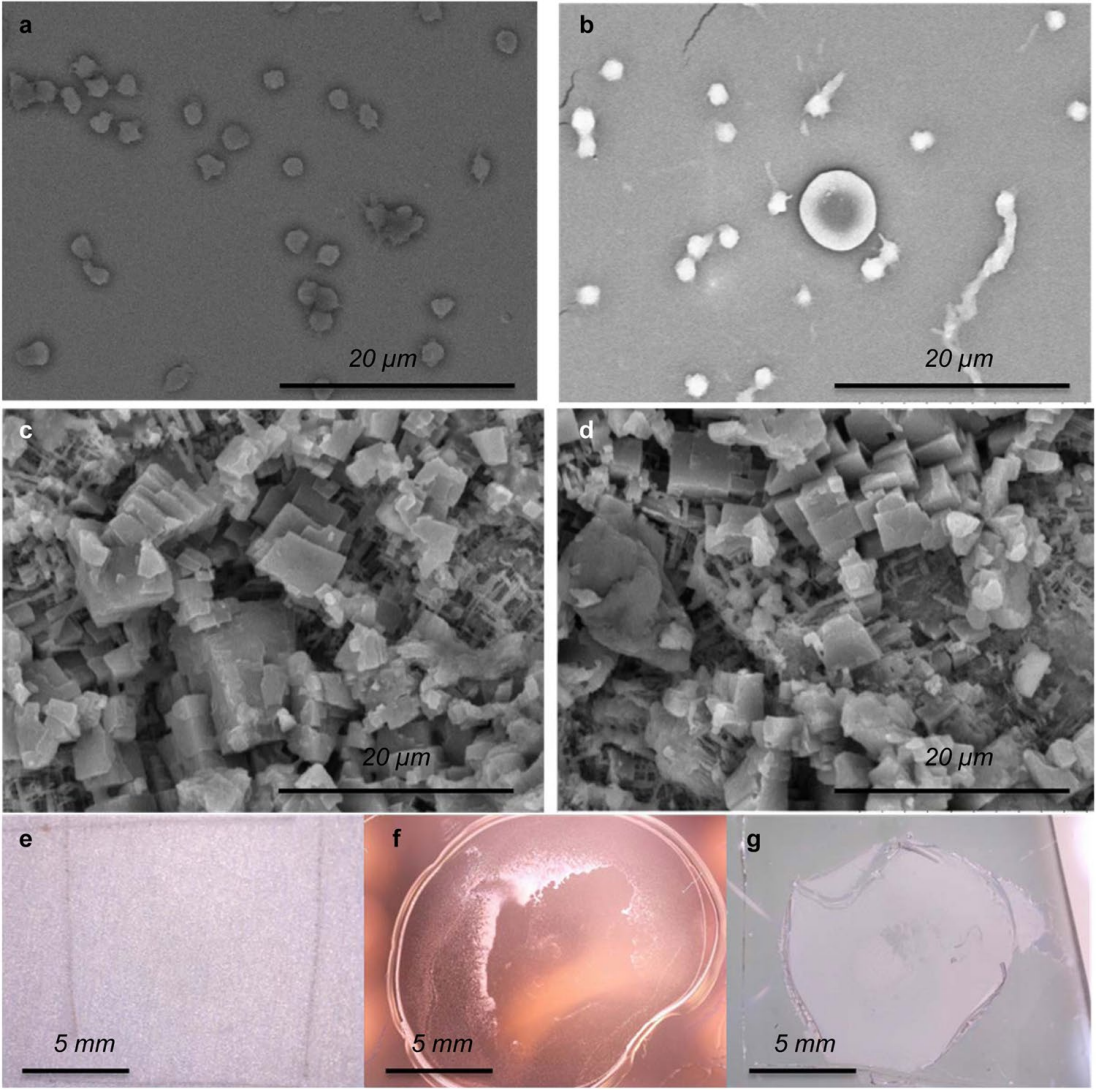
Platelet adhesion test. SEM micrographs of samples after 20-min incubation in platelet rich plasma on (**a**) smooth titania and (**b**) smooth PDMS (**c**) superhydrophobic PDMS/titania before incubation (**d**) superhydrophobic PDMS/titania after incubation. Scale bars are 20 µm in (**a–d**). Images of surfaces after incubation in platelet rich plasma on (**e**) superhydrophobic PDMS/titania surface, (**f**) smooth titania, (**g**) smooth PDMS. Scale bars are 5 mm in (**e–g**).

Figure 4 shows the SEM images of the platelet adhesion test on smooth titania (Figure 4a), smooth PDMS (Figure 4b) and nanostructured PDMS/titania before (Figure 4c) and after (Figure 4d) the test. The test revealed that there were no adhered platelets on the superhydrophobic surface while there were many adhered platelets on both the smooth titania and the smooth PDMS substrate. The platelets on the control surfaces are aggregated and show dendritic extensions, indicating activation. It has been reported that the robustness of the Cassie state is a key determinant of blood repellency on superhydrophobic surfaces^31^ as the plastron greatly reduces physical contact between the platelets and the surface. The plastron thickness was measured to be 70 µm using confocal microscopy (Supplementary Information and Supplementary Figure S4). The Cassie state on our tube surfaces is robust because the plastron layer is very thick due to the thickness of the porous layer. High stability of the plastron under water storage was investigated previously^42^.

The surfaces also present hierarchical structure where the nanostructures are smaller than the size of platelet, which has been shown to lead to blood repellent surfaces^35^. Figure 4e shows that after incubation with a blood droplet and washing gently with water, the superhydrophobic surface does not show any residues (the blood droplet resided inside the square shaped markings carved to the surface to mark the location). In contrast, the control titania (Figure 4f) and smooth PDMS (Figure 4g) surfaces show large surface deposits composed of adherent platelets and residues of extracellular matrix, e.g. fibrin remaining after the repeated additions of platelet reach plasma (PRP).

## Conclusions

With a flexible and durable superhydrophobic tube, both drag reduction and droplet manipulation of water and blood droplets was achieved. The tubes were fabricated by scalable top-down process. The resulting hybrid PDMS/titania tube is bendable, robust, semi-transparent and highly superhydrophobic. The inner diameter of the tubes can be varied from micrometer to centimeter scale, depending on the template tube. The superhydrophobicity of the inner wall is demonstrated by 68% drag reduction in continuous flow and up to 1000-fold increase in acceleration of sliding droplets. The tubes are blood repellent. As a prototype, we showed how a 10 cm long tube with 4 mm of diameter to transfer 200 µL of human blood with no blood staining and no platelet adhesion. The same method can also be used to fabricate wires and tubes with outer surfaces rendered superhydrophobic. Our superhydrophobic tubes can open up new applications in blood related bio-medical devices and blood vessel implants^43–46^.

## Methods

The following section provides description of fabrication process and characterization methods used in this study. Written informed consent was obtained from the subject for participation for all the blood samples. The experiments were carried out in accordance with institutional guidelines and safety regulations which were approved by Helsinki University in Finland, for handling of biological material under supervision of a licensed physician. The ethical permit is issued by the Operative Ethics Committee for the Hospital District of Helsinki and Uusimaa.

### Fabrication Process

The non-lithographic fabrication process was started with aluminum alloy 6061 tubes from OnlineMetals USA. Wet etching process was used to produce nanostructures on aluminum tube as a template. 75 min etching was done in a 1 M HCl solution prepared from 37% HCl Sigma Aldrich. After 2 min rinsing the wafer was dried by nitrogen gun. 20 nm thick titania was deposited on it using ALD. Shortly, titania 20 nm ± 1% thick at 300 °C temperature was deposited in a Beneq TFS-500 reactor using titanium chloride (TiCl_4_) as a metal precursor and water as a precursor for oxidation. The pressure in the reactor was kept at about 4 Torr. Nitrogen was used as a carrier gas and to purge reaction gases from the reactor during each reaction half cycle. 400 cycles of 1500 ms precursor pulses and 1500 ms purge pulses (the same for both precursors) were used. Long purge and pulse time is needed for fully cover the 3D high aspect ratio nanostructures of template. Then a five millimeter thick mixture of 10:1 monomer to crosslinking agent ratio PDMS (Sylgard 184) was cured for 2 hours at 50 °C between the aluminum tubes. After curing the PDMS, the inner aluminum tube was removed using 12 M HCl while the outer aluminum tube was protected by scotch tape. The titania layer was transferred to the structured PDMS surface in this step. After the sacrificial release of the nanostructures, the PDMS/titania replica was separated from the outer aluminum tube by mechanical peeling. Separation followed by washing and rinsing with DI water. The PDMS/titania tube was then annealed in an oven for 1 hour at 50 °C. The inner surface of the tube was superhydrophobic after annealing.

XPS measurement was performed to confirm the existence of titania layer after replication to PDMS. A Quantera SXM spectrometer made by Physical Electronics was used. The radiation was provided by a monochromatized Al Ka (1486.6 eV) X-ray source, operated at emission power of 25 W and at acceleration voltage of 15 kV.

### Sliding Droplets

An in-house built goniometer was used to tune the tilting angle of the tubes to measure the acceleration of sliding droplets inside the tilted tubes. A camera used to record a video of the sliding droplets. The acceleration (a = 2Lt^−2^) was calculated using the time (t) that takes for droplet to pass through the tube with length (L). Figure S2 shows a photo of the setup with a tube mounted on 40° tilted angle. A 35 µL droplet was used for all the measurements. The reported acceleration data is an average of three measurements for each tilted angle.

### Confocal Microscopy

Figure S4a,b shows confocal microscopy images from the bottom of the porous area and the water-porous area interface on the PDMS/titania sample respectively. Schematic of the measurement setup is shown in Figure S4c. Acetone soluble fluorescent dyes (Nile Red from Sigma Aldrich) were used to dye the sample. A thin soda-lime glass microscope slide was used to avoid wetting the objective. The plastron thickness was measured by focusing on “a” and “b” in Figure S4c for different points of the surface. The result of 70 µm is an average of 5 measurements.

### Blood and platelet adhesion tests

For the experiments using full blood on surfaces and in tubes, blood from healthy volunteers was drawn by venipuncture to 6.0 ml BD Vacutainer Plus K2EDTA tubes (367863, BD, Becton, Dickinson and Company, Franklin Lakes, NJ). After collection, the tubes were immediately gently mixed by repeated inversion 5–6 times. For the droplet experiments venous blood was drawn from healthy volunteers to 1.8 ml citrate collection tubes (363047, BD Vacutainer, 0.109 M Buffered Trisodium Citrate). Tubes were immediately gently mixed by inversion repeated 3–4 times. The samples were then centrifuged for 20 min at 100 g (no brake, room temperature). Supernatant platelet-rich plasma (PRP) was carefully removed and transferred into 5 ml tubes (Eppendorf 5 ml tubes, 0030122321). The collection tubes were stored at room temperature. PRP aliquots of 10 µl (1:2 dilution in 0.01% D-glucose, 5 µM CaCl_2_•2H_2_O, 98 µM MgCl_2_•6H_2_O, 540 µM KCl, 126 mM NaCl, 14.5 mM Tris pH7.4) were pipetted onto different surfaces (drops) three drops/material surface. The drops of PRP on the surfaces were incubated at + 37 °C under a moist environment for 20 minutes. All drops were then removed and replaced with fresh drops by pipetting. Each site underwent three incubation-drop removal-PRP addition cycles. After completion of the cycles, 10 µl of 10% neutral buffered formalin solution (Sigma HT501128) was added to each drop site. Formalin was replaced with same volume fresh formalin solution after overnight incubation. Imaging was performed the following day. The samples were gently washed by immersing in water for five seconds three consecutive times. The samples were then dried under atmosphere for 2 hours, coated with a thin layer of gold (SCD 050, Bal-Tec) and imaged by SEM (TM-1000, Hitachi, Japan).

### Data availability

All data generated or analysed during this study are included in this published article (and its Supplementary Information files).

## Electronic supplementary material

Video S1

Video S2

Supplementary Information
