# Supplementary material for: Water and Blood Repellent Flexible Tubes
Source: Sci Rep. 2017 Nov 22;7:16019. doi: 10.1038/s41598-017-16369-3 (PMC5700071; doi:10.1038/s41598-017-16369-3)
Supplement: Supplementary file 3 — Supplementary Information [file 41598_2017_16369_MOESM3_ESM.pdf]

Water and Blood Repellent Flexible Tubes

Sasha Hoshian[1,2], Esko Kankuri[3], Robin H. A. Ras[4], Sami Franssila[1], Ville Jokinen[1]**

[1] Sasha Hoshian*, Dr. Ville Jokinen*, Prof. Sami Franssila
Department of Chemistry and Materials Science
Aalto University School of Chemical Engineering, Espoo, Finland
[2] Sasha Hoshian
Brigham and Women's Hospital, Harvard Medical School, Cambridge, MA 02139, USA
[3] Dr. Esko Kankuri
Faculty of Medicine, Department of Pharmacology
University of Helsinki, Helsinki, Finland
[4] Prof. Robin H. A. Ras
Department of Applied Physics
Aalto University School of Science, Espoo, Finland
*: Corresponding author
Sasha Hoshian (E-mail: sasha.hoshian@aalto.fi)
Dr. Ville Jokinen (E-mail: ville.p.jokinen@aalto.fi)
Aalto University, Tietotie 3, Espoo, Finland 02150

Supplementary Information

Replication of PDMS nanostructures with and without ALD titania

The successful replication of thick layer of nanostructures from aluminum template to PDMS is strongly relies on the ALD titania layer. The replicated process was done with and without using ALD titania. Figure S1a shows the XPS data of the replicated sample with ALD titania. Data confirms the existence of titania layer on PDMS nanostructures after replication. SEM micrographs on replicated samples with and without using ALD titania are shown in Figure S1b-c respectively. It is clearly visible that the ALD-protected structures have much sharper features which bear close similarity to the template. In contrast, the features of the replicated sample without ALD titania are much more rounded because the nanoscale structures are not accurately replicated.

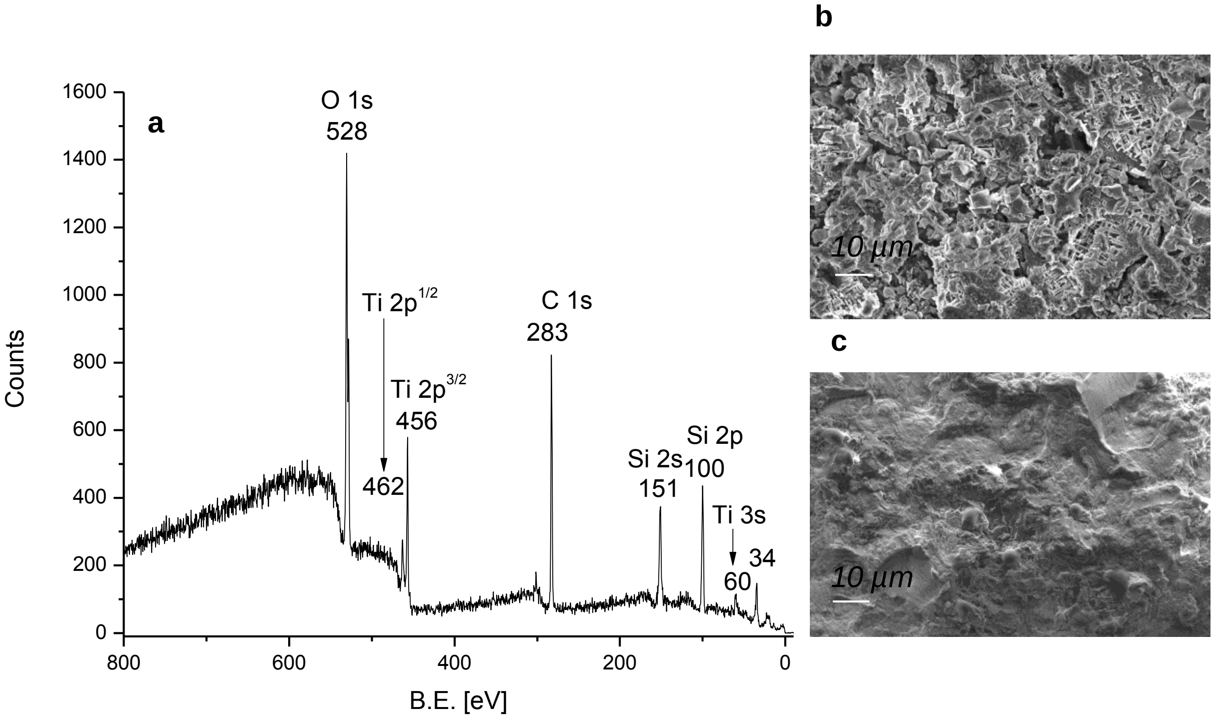

Figure S1. a) XPS data of the PDMS/titania sample after replication confirms the existence of titania. b) SEM micrograph of nanostructure PDMS replicated using ALD titania and c) without using ALD titania. Scale bars are 10 µm.

Sliding Droplets

An in-house built goniometer was used to tune the tilting angle of the tubes to measure the acceleration of sliding droplets inside the tilted tubes. A camera used to record a video of the sliding droplets. The acceleration ($a = 2Lt^{-2}$) was calculated using the time (t) that takes for droplet to pass through the tube with length (L). Figure S2 shows a photo of the setup with a tube mounted on 40° tilted angle. A 35µL droplet was used for all the measurements. The reported acceleration data is an average of three measurements for each tilted angle.

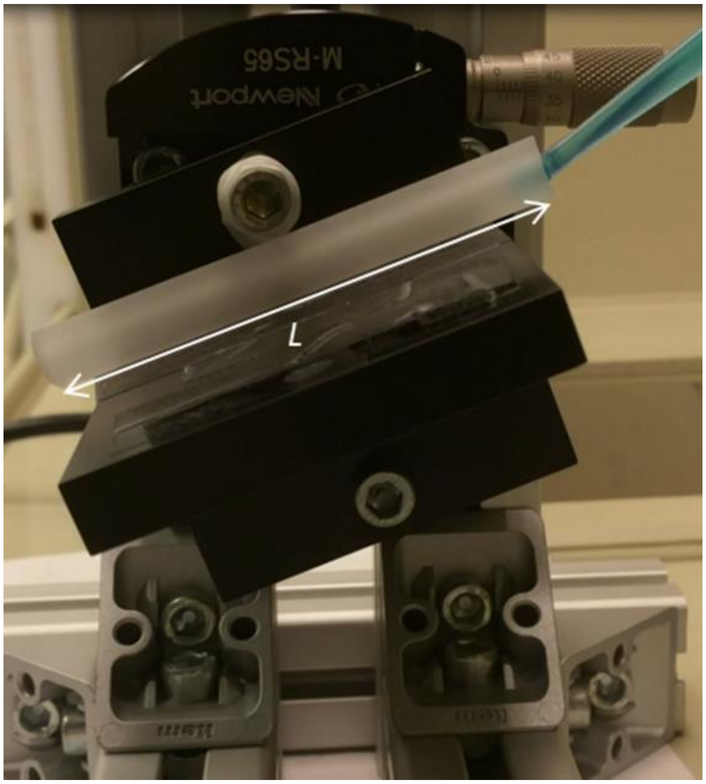

Figure S2. The sliding droplet measurement setup. The length of the tube (L) is 12 cm.

Drag Reduction

In the case of water flowing in a superhydrophobic tube, flow rate and the δ/r ratio are important factors where δ is the plastron thickness and r is the tube radius. We compared the flow rates in a smooth PDMS/titania control and superhydrophobic PDMS/titania tubes to show the effect of superhydrophobicity. For superhydrophobic PDMS/titania samples, the plastron thickness was measured to be 70 μm on a planar surface using confocal microscopy. The plastron thickness is less than the thickness of porous area (l) because of the water penetration. Flow rate inside both superhydrophobic and control tubes were measured with a setup similar to reference 40. Schematic of the setup is illustrated in Fig. S3a. A tube was connected to a water tank and the time to collect 150 gr of water was measured using a stopwatch. The volume of tank was chosen to be much larger than the

volume of the tube to ensure that the water level reduction in the tank during the experiment was negligible. In this setup, laminar flow is expected for the tube diameters of 2 mm and 4 mm when the height of air-water interface from the tube center (h) varies from 1-9 cm. Figure S3b shows the flow rate in superhydrophobic and control tubes with 2 and 4 mm inner diameters. The flow rate increases linearly by increasing h for all the tubes. Superhydrophobic tubes show higher flow rate compared to their control for both tubes. The increase of the flow rate in a superhydrophobic tube is due to the existence of plastron layer, which causes the friction reduction between, solid and liquid in the tube. In the control tube a Poiseuille flow with no-slip (zero velocity at the interface) boundary condition (Fig. S3c) is expected while in the superhydrophobic tube an almost plug flow caused by trapped air pillow under thick porous area (Fig. S3d) is dominant. Although the 4 mm diameter tube shows higher flow rate compare to 2 mm diameter, drag reduction was more significant in the 2 mm diameter tube. The drag reduction was calculated using equation S1.

$$\%\, Drag\ reduction = 100 \times \frac{\mathrm{R(control)} - \mathrm{R(SHB)}}{\mathrm{R(control)}} \qquad \text{(equation S1)}$$

Where R(control) and R(SHB) are the resistance of the smooth PDMS/titania control tube and superhydrophobic PDMS/titania tube respectively. The resistances of tubes were calculated using equation S2.

$$R = \frac{\Delta \mathrm{P}}{\mathrm{Q}} = \frac{\rho g h}{\mathrm{Q}} \qquad \text{(equation S2)}$$

Where ΔP is the pressure difference between the tube center and water-air interface in the tank, Q is the volume flow rate, ρ is the water density at room temperature, g is the gravitational acceleration and h is the height of the water-air interface from the tube center. Figure S3e shows the percentage of drag reduction as a function of the Reynolds numbers for the tubes. A maximum of 68% drag reduction was

observed for the 2 mm diameter tube in low Reynolds numbers while for the 4 mm diameter tube the maximum was 38%. The δ/r ratio is bigger for the 2 mm diameter tube, which explains the higher drag reduction.

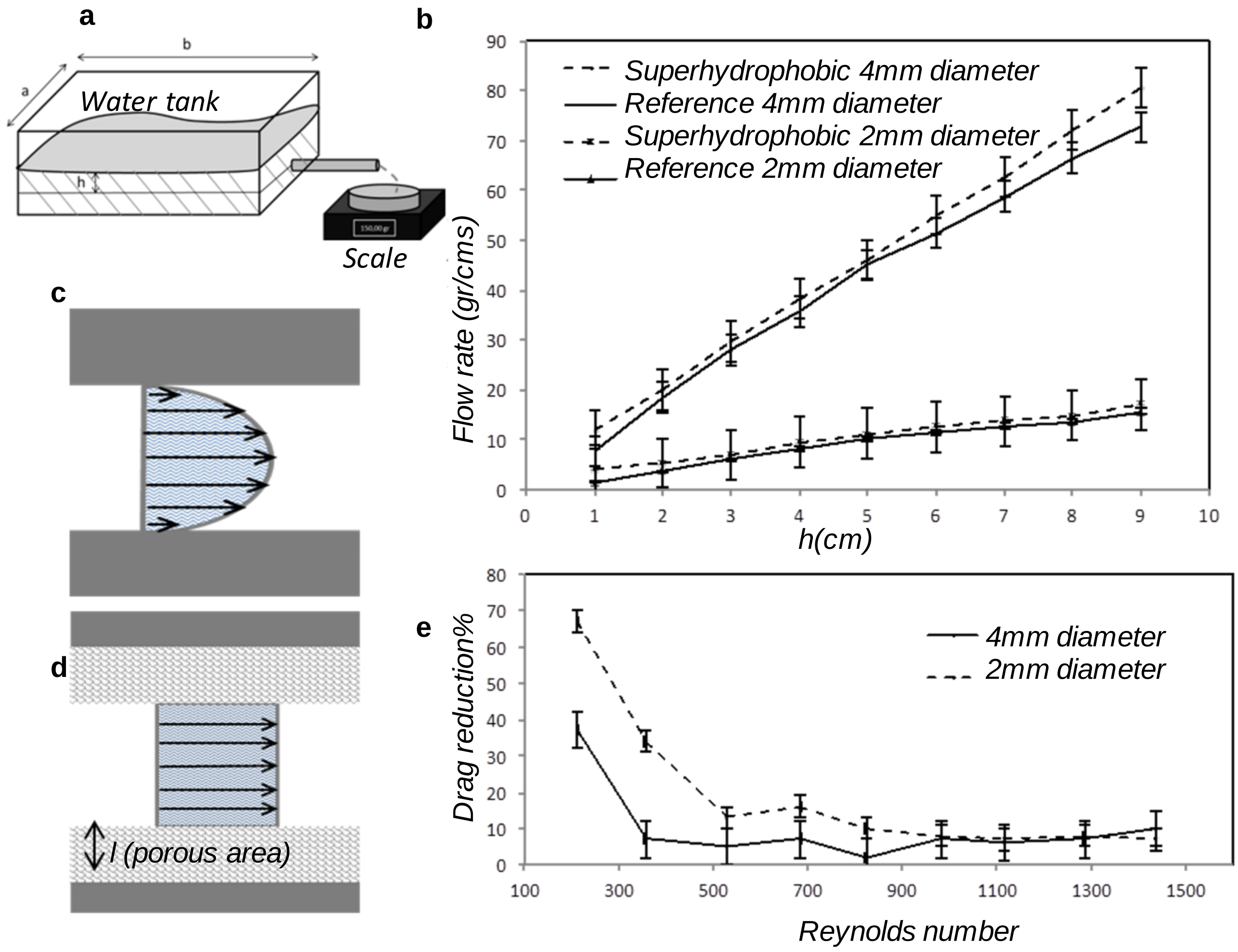

Figure S3. a) Schematic of flow measurement setup by weighting. b) Flow rate measurement for 2 and 4 mm diameter control (lines) and superhydrophobic (dots) tubes. c) Poiseuille flow with no-slip (zero velocity at the interface) boundary condition in a control smooth tube, d) Plug flow causing by trapped air pillow under thick porous area. e) Drag reduction as a function of Reynolds number.

Confocal Microscopy

Figure S4a-b shows confocal microscopy images from the bottom of the porous area and the water-porous area interface on the PDMS/titania sample respectively. Schematic of the measurement setup is shown in Fig. S4c. Acetone soluble fluorescent dyes (Nile Red from Sigma Aldrich) were used to dye the sample. A thin soda-lime glass microscope slide was used to avoid wetting the objective. The plastron thickness was measured by focusing on "a" and "b" in Fig. S4c for different points of the surface. The result of 70μm is an average of 5 measurements.

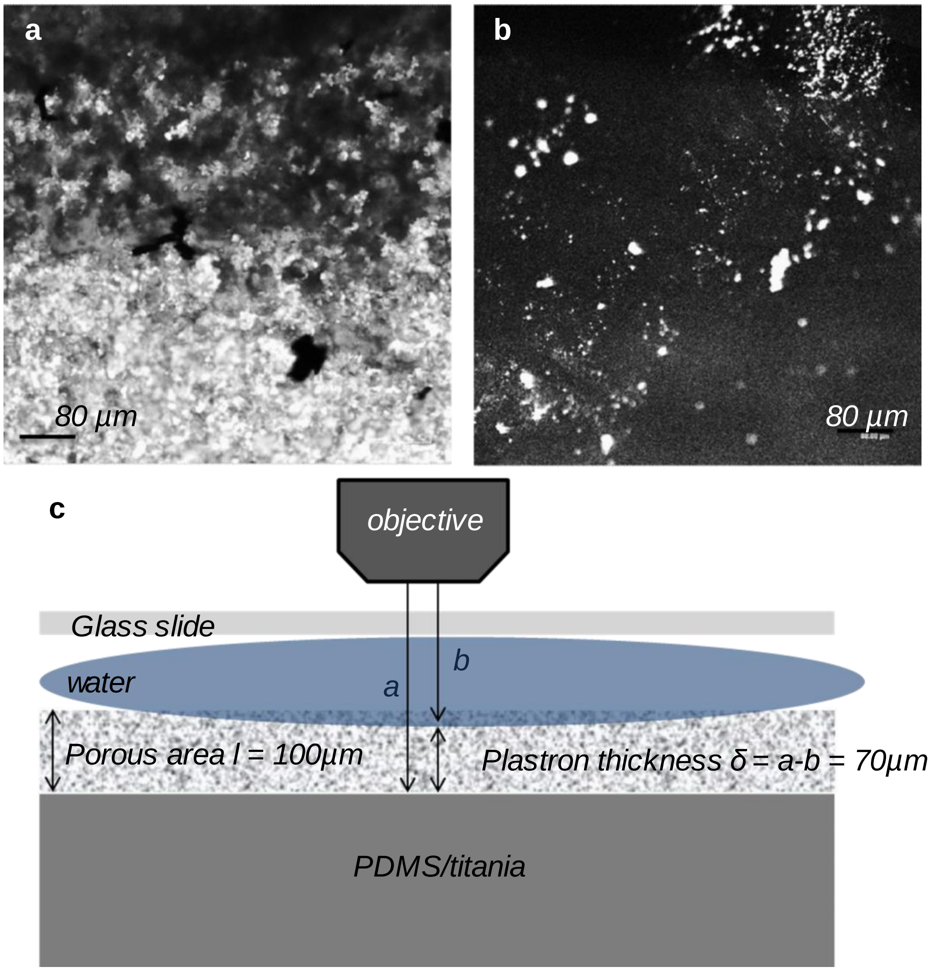

Figure S4. Plastron thickness measurement. Confocal microscopy images a) the bottom of porous area, b) the water-porous area interface, c) Schematic of the measurement setup.
